# Estimating Hourly Concentrations of PM_2.5_ across a Metropolitan Area Using Low-Cost Particle Monitors

**DOI:** 10.3390/s17081922

**Published:** 2017-08-21

**Authors:** Nadezda Zikova, Mauro Masiol, David C. Chalupa, David Q. Rich, Andrea R. Ferro, Philip K. Hopke

**Affiliations:** 1 Institute for Environmental Studies, Faculty of Science, Charles University, Prague 12801, Czech Republic; nada.zikova@natur.cuni.cz; 2 Center for Air Resources Engineering and Science, Clarkson University, Potsdam, NY 13699, USA; mmasiol@clarkson.edu; 3 Department of Public Health Sciences, University of Rochester School of Medicine and Dentistry, Rochester, NY 14642, USA, David_Rich@urmc.rochester.edu; 4 Department of Environmental Medicine, University of Rochester Medical Center, Rochester, NY 14642, USA; David_Chalupa@urmc.rochester.edu; 5 Department of Civil and Environmental Engineering, Clarkson University, Potsdam, NY 13699, USA; aferro@clarkson.edu

**Keywords:** particulate matter, low-cost monitors, spatial variability, hourly concentrations

## Abstract

There is concern regarding the heterogeneity of exposure to airborne particulate matter (PM) across urban areas leading to negatively biased health effects models. New, low-cost sensors now permit continuous and simultaneous measurements to be made in multiple locations. Measurements of ambient PM were made from October to April 2015–2016 and 2016–2017 to assess the spatial and temporal variability in PM and the relative importance of traffic and wood smoke to outdoor PM concentrations in Rochester, NY, USA. In general, there was moderate spatial inhomogeneity, as indicated by multiple pairwise measures including coefficient of divergence and signed rank tests of the value distributions. Pearson correlation coefficients were often moderate (~50% of units showed correlations >0.5 during the first season), indicating that there was some coherent variation across the area, likely driven by a combination of meteorological conditions (wind speed, direction, and mixed layer heights) and the concentration of PM_2.5_ being transported into the region. Although the accuracy of these PM sensors is limited, they are sufficiently precise relative to one another and to research grade instruments that they can be useful is assessing the spatial and temporal variations across an area and provide concentration estimates based on higher-quality central site monitoring data.

## 1. Introduction

Ambient particulate matter (PM) has a variety of adverse effects on human health [1]. In the United States, PM concentrations are measured at many fixed air quality stations in monitoring networks managed by state, local, and tribal agencies to assess compliance with National Ambient Air Quality Standards (NAAQS) for PM_2.5_, PM_10_, and other criteria pollutants. Solomon et al. [2] provide a review of regulatory monitoring methods, which are designated as Federal “Reference” or “Equivalent Methods” (FRM and FEM, respectively) in accordance with Code of Federal Regulations (40 CFR Part 53). FRM and FEM involve the use of scientific-grade instruments, which are generally expensive to purchase, may be large, have substantial power demands, and require periodic maintenance and/or handling by operators. These limitations typically mean that a limited number of stations are deployed within major cities, and there are few stations located in remote areas and close to hotspots (e.g., industrial plants or high-trafficked roads) [3] except for current efforts to monitor the near-road environment at a limited number of sites [4]. In urban areas, urban background stations are placed in areas broadly representative of the city-wide pollution. However, the resulting limited spatial and temporal resolution may be a limitation for epidemiological studies, which aim to represent the inhalation exposure of people living across large areas with varying pollutant levels [5]. Lin et al. [6] recently showed that hourly peak PM concentrations reflect health impacts better than daily averages. Thus, it would be useful to obtain spatially resolved information on an hourly basis to provide additional information for epidemiological studies.

The use of recently developed low-cost monitoring instruments (prices in 100 s of USD) can be a useful option to improve the temporal resolution and spatial coverage of hourly PM data [7,8]. These low-cost instruments are physically small and light, have low power demands, and require less handling and maintenance compared to scientific-grade FRM and FEM instruments. Consequently, many sampling points can be monitored over wide areas to better elucidate the spatial variation of the air pollutants, for example to identify hotspots in a polluted environment [9], to estimate personal exposure [10], etc. However, low-cost monitors do not meet rigid performance standards and, therefore, have limitations [7,11,12]. Because these low-cost monitors are being deployed by both researchers and the general public, there is a need to assess both the advantages and the limitations of low-cost instruments when determining their usefulness for large-scale studies [13].

Recently, several studies assessing low-cost PM monitors have been published for measuring occupational PM in a laboratory setting (e.g., [14]), laboratory and ambient comparisons (e.g., [8,15]), and field comparisons (e.g., [11,16]). The number of such studies, however, is low compared to the number of low-cost devices produced globally, as many low-cost PM monitors are now on the market and the manufacturers are not required to provide testing to validate them [13].

In previous studies, Manikonda et al. [17] and Zikova et al. [18] evaluated the performance of several low-cost PM monitors, with further testing on one of the monitor types, the Speck (Airviz Inc., Pittsburgh, PA, USA). These monitors were tested under laboratory and field conditions and compared to reference scientific-grade instruments in side-by-side collocated measurements. In the present study, extended duration field measurements were performed to obtain information on monitors’ consistency, stability, and durability of the sensing elements. The Speck units were deployed outdoors over two winter sampling campaigns to estimate the spatial variability of urban and suburban PM_2.5_. This study describes the results of two sampling campaigns and discusses the utility of low-cost instruments to determine the spatial variability of hourly PM pollution across a large urban area for consideration of their use in future studies. 

## 2. Methods 

### 2.1. Measurement Periods

The measurements were conducted during two heating season sampling campaigns across Monroe County, NY, USA covering the period of late fall to early spring. These “heating seasons” represent periods when ambient PM concentrations are affected by the emissions from space heating [19], including residential wood combustion [20,21]. The first measurement period started in early December 2015 (10th to 12th December) and ended in early April 2016 (4th to 10th April) since it took several days to deploy and retrieve the monitors. The second measurement period started at the end of October 2016 (27th October to 22th November) and lasted until early April 2017 (2nd to 5th April). The number of data points in each period is presented in Table 1 (with the names starting with V denoting measurements during the first period, and names starting with P denoting measurements during the second year).

### 2.2. Instruments

During the two periods, PM_2.5_ measurements were made outdoors using low-cost PM monitors at approximately 25 residential locations per year and at the NY State Department of Environmental Conservation (DEC) air monitoring station in Rochester, NY using a tapered element oscillating microbalance (TEOM) that has been designated as a Federal Equivalent Method for determining compliance with the PM_2.5_ National Ambient Air Quality Standard (NAAQS).

#### 2.2.1. Speck Monitors

The Speck air quality monitor (Airviz Inc., Pittsburgh, PA, USA) uses an infrared LED-based Syhitech DSM501A dust sensor. This sensor works on a light scattering principle, estimating the mass concentration from the detected scattered light, and measures particle concentrations in the size range of 0.5 to 3 µm. A small fan pulls the air into the sensor and the concentrations are measured at an adjustable time resolution from 30 s to 4 min. The data are stored in the internal memory and directly uploaded to the manufacturer’s server when connected to a Wi-Fi network. The instrument was previously tested with several other low-cost monitors under laboratory conditions [17] and under residential indoor and outdoor ambient conditions [18]. The results of these previous studies showed a linear response [17], high reproducibility of results, precision of 12% for outdoor data, and a positive bias [18].

From the previous field comparison [18], a limit of detection (LOD) was assessed to be 10 µg/m^3^ for raw PM concentrations. Thus, from the measured data in this study, two datasets were derived. The first dataset contains all the originally measured values. In the second dataset, the values below LOD were replaced by the LOD/2 value, i.e., 5 µg/m^3^. The percentage of data above LOD is reported in Table 1. From both datasets (LOD corrected and non-corrected), hourly and daily means were calculated (mean values were considered only if at least 75% of data were available, i.e., 45 min for each hourly average, and 18 h for each daily average) and a date- and time-matched dataset from the Speck data was created.

Each outside Speck monitor was placed in an outdoor housing (Figure S1 in the Supplementary Materials) protecting the instrument from water and extreme cold. Heat from a 6-W light bulb was included in the housing. No negative influences of the housing on the bias, correlation with the reference instrument, or precision were observed during a comparison made between the two periods. The instruments were checked before, during (mid-term between January and March), and after each sampling campaign. During these checks, the housing inlets were cleaned, the sensor was checked for malfunctions or power outages, and the zero level was checked. The data were downloaded using a USB connection to a computer if Wi-Fi was unavailable. During the 2016–2017 measurement period, several monitors were replaced during the mid-term check with new units to allow the recovery of the data by the manufacturer. In such locations, the data were corrected separately for each Speck, and the corrected data were later merged into a single dataset.

A field comparison was performed after each measurement period where all the Speck monitors were collocated with a Grimm 1.109 Aerosol Spectrometer (Grimm Technologies, Douglasville, GA, USA). This collocation test was carried out both indoors and outdoors at a selected residence with an operating wood burning appliance in Potsdam, NY. Bias corrections were calculated from three days of field data by comparing the Speck monitors and Grimm instrument. The corrections were calculated as a ratio of the mean PM_2.5_ concentration measured by the Grimm, and the mean concentration measured by a Speck unit during the collocation experiment [18].

For the first measurement period, the data from the outdoor collocation campaign were used. However, for the second period, the PM concentration during the outdoor collocation and the associated correlations between the Speck units and Grimm were low probably because of the very low ambient PM concentrations. Thus, the indoor correction factors, which were based on higher mean instrument correlation, were chosen to normalize the data. For both periods, the bias corrections for indoor and outdoor were similar (R^2^ > 0.9). The bias corrections (ranging from 0.15 to 0.96 with mean value of 0.36, Table 1) were applied to the data after the LOD correction and prior to the analyses.

#### 2.2.2. Tapered Element Oscillating Microbalance (TEOM)

A Thermo Scientific (Waltham, MA, USA) 1405-DF TEOM™ at the Rochester NYS DEC site was used as a reference measurement. The TEOM is a Federal Equivalent Method (FEM) for the PM_2.5_ measurements. Data were downloaded from New York State Air Quality website (http://www.nyaqinow.net/) in 1-h time increments for both periods and compared to the hourly averages calculated from the collocated Speck monitor. Twenty-four-hour averages were calculated if at least 75% of data was available during that day. The 24-h averages were compared to daily averages from Speck monitors located across Monroe County.

### 2.3. Study Area

**Figure 1:**
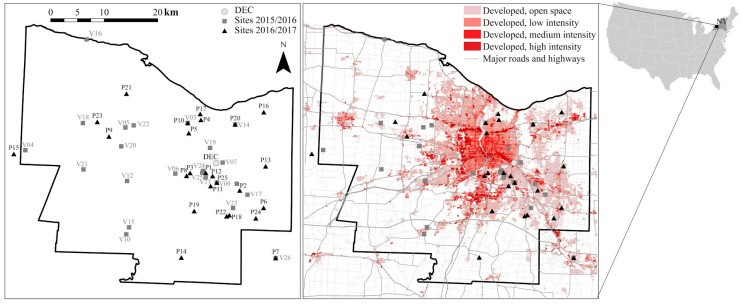
Speck units’ locations across the Monroe County, NY, during the two heating seasons. Background: Developed class defined in the USGS National Land Cover Database 2011.

The measurements were made in Monroe County, NY, which includes the city of Rochester (43°9′26″ N, 77°36′23″ W, 154 m a. s. l.), at 27 and 26 residential locations during the 2015–2016 and 2016–2017 campaigns, respectively (Figure 1). The total land area of the county is ~1700 km^2^. In 2015, there were estimated to be 749,600 inhabitants [19]. The major primary emissions sources include residential wood smoke, road traffic, other residential and commercial heating, and a few industrial sources [20,21]. Road traffic includes major routes carrying traffic to and from downtown (e.g., Route 96) as well as Interstate Highways I-90, I-390, I-490, and I-590. Natural gas is the primary fuel for domestic and commercial heating, with oil accounting for most of the remaining homes and buildings. Wood combustion in most Monroe County residences is recreational. However, Wang et al. [21] found that wood smoke can represent up to 30% of the winter-time PM_2.5_ concentrations. Industrial emissions were largely dominated by a coal-fired cogeneration plant (Eastman Kodak complex) located in an area ~6 km NW from downtown, but its production substantially decreased during recent years following the decline of film production. Other sources may be different types of off-road transport (e.g., diesel rail, shipping on Lake Ontario, and emissions from the airport). Regional transport brings polluted air masses from Ontario, the Ohio River Valley, and the highly populated east coast of the United States [22].

### 2.4. Measurement Sites

Twenty-five units were deployed in the yards of selected volunteers across Monroe County during the two seasons. One more Speck unit was co-located at the DEC site in Rochester (Figure 1), where FRM and FEM instruments were available as well as additional data on air quality (CO, O_3_, SO_2_, and NO_y_ concentrations, downloaded from New York State Air Quality website http://www.nyaqinow.net/). 

It was not possible to use a spatially optimal approach, as suggested by Kumar et al. [7], since the study depended on volunteers and another part of this study required indoor measurements. The number of available instruments limited the number of volunteers. The study volunteers were recruited mostly from employees of the University of Rochester. Inclusion criteria for the study were that homes had a wood-burning appliance or were in areas with wood smoke pollution based on the volunteers’ own observations. 

All measurement sites were located near single-family houses, preferably on the windward side of the house based on the prevailing wind direction. The distance between the buildings and the Speck units varied between 2 and 25 m, and the instruments were placed about 1.5 m above the ground. The most common locations were fences, open porches, patios, and gardens using poles to elevate the monitors. These units require connection to electricity. Most Speck units were not Wi-Fi-connected during the sampling campaigns due to the distance of the measurement sites from the Wi-Fi routers in the houses.

### 2.5. Meteorological Data

Meteorological records of precipitation, snowfall, temperature, relative humidity, dew point, wind speed and direction, and weather types were retrieved from the National Climate Data Center for the Greater Rochester International Airport (KROC) as hourly data to meteorologically characterize the measurement period. The airport lies between 2 km and 29 km from the monitoring locations, and 10 km from the closest NY State DEC air monitoring station.

During the 2015–2016 measurement period, the average temperature was 1.1 °C, with minimum and maximum hourly temperatures of −23.9 and 23.9 °C, respectively. During the 130 days of the campaign, 375 mm of precipitation was measured. During the 2016–2017 measurement period, the average daily temperature was 1.8 °C, with minimum and maximum hourly temperatures of −15.6 and 22.8 °C, respectively. During the 162 days of this campaign, 542 mm of precipitation was measured. Thus, the weather during the two measurement campaigns was somewhat different, with the second heating season being warmer and wetter than the first.

### 2.6. Data Analyses

#### 2.6.1. Wilcoxon Signed-Rank Test

Speck and TEOM data were compared using non-parametric Wilcoxon signed-rank test [23] to test if the paired data were selected from populations having the same distribution. The paired version of wilcox.test script, package ‘stats’, was run in R (version 3.1.2). Data during each measurement period were considered separately.

#### 2.6.2. Correlation Analysis

Hourly and daily averages calculated from each Speck instrument across the sampled domain were compared to the TEOM values and presented with respect to the distance of the given Speck from the TEOM irrespectively of the compass direction from the TEOM. The one-minute data were not considered since the uncertainty in PM_2.5_ concentration on a 1-min scale were large and calculating one-hour values averages out random noise. Pearson correlation coefficients were calculated. The two measurement periods were evaluated separately. Speck monitor data were compared to the TEOM data from only the corresponding period. The analysis of the correlation on the distance from the TEOM was performed using the data from both periods.

Correlation coefficients were determined between each pair of Speck units resulting in a correlation matrix based on hourly and daily data. The correlation matrix was calculated for each measurement period separately, as both concentrations and locations of Speck monitors differed.

#### 2.6.3. Coefficient of Divergence

The pairwise coefficients of divergence (COD) [24] were calculated as the relative spatial variability assessment. The COD between two measurements is defined as:

(1) $$COD_{jk}=\sqrt{\frac{1}{p}\overset{p}{\underset{i=1}{\sum }}{\left[\left(x_{ij}-x_{ik}\right)/\left(x_{ij}+x_{ik}\right)\right]}^{2}}$$

where *x_ij_* is the *i*-th averaged concentration measured by one of the units at the location *j*, *j* and *k* are two different measurements (done at different locations), and *p* is the number of observations. “A COD value equal to zero means the concentrations are identical at both sites, while a value approaching one indicates substantial heterogeneity. COD values greater than approximately 0.20 indicate relatively heterogeneous spatial distributions [25,26].” [19]. 

The COD values for Speck units and for the Specks and TEOM were also calculated based on hourly and daily averages. 

#### 2.6.4. Spatial Interpolations

The PM concentrations within urban areas are strongly influenced by the spatial distribution of anthropogenic activities, topography, and meteorology. Thus, one or few sampling sites complying with FRM and/or FEM are not likely to be sufficient to describe the spatial variation of PM across large urban areas. Spatial interpolations have been extensively used to model the small-scale intra-urban variations of air pollution (e.g., [19,27,28]). The hourly and weekly averages (calculated over the whole measurement periods), were interpolated using inverse squared-distance interpolation (IDW) with the weight of power of 2, i.e., the influence of neighboring points is diminished as a function of increasing distance *d* as of *d*^2^ [29]:

(2) $$x_{gc}=\left[\underset{i}{\sum }\left(x_{i}/d_{i}^{2}\right)\right]/\left[\underset{i}{\sum }\left(1/d_{i}^{2}\right)\right]$$

where *x_gc_* is the interpolated value at a grid cell, *x_i_* is the value measured at the *i*-th nearest neighbor, and di is the distance from the grid cell to the *i*-th neighbor. The IDW method was chosen for its simplicity, method not requiring data pre-modeling or any assumption fulfillment. The interpolated maps were created using R and a series of packages, including ‘rgdal’ and ‘spatstat’. The resulting maps were animated into a video to show the spatial-temporal variations of PM concentrations measured by the Specks and are available in the supplemental information.

#### 2.6.5. Conditional Bivariate Probability Function

The conditional bivariate probability function (CBPF; [30]) is based on conditional probability function (CPF, [31]) defined as:

(3) $$CPF=m_{i}/n_{i}$$

where *m_i_* is the number of samples in the wind sector *i* with mixing ratios greater than a given concentration, and *n_i_* is the total number of samples in the same wind sector. The CBPF also takes the wind speed into account by creating a continuous surface calculated through modelling using smoothing techniques. As a threshold, the 50th percentile was considered, and the CBPF was calculated both from Speck data and from TEOM data in each of the measurement periods.

## 3. Results and Discussion

### 3.1. Data Availability

The data completeness was high for most of the units. Of the 50 measurement locations, 46 had data completeness of over 90%, and 31 had 100% data completeness (Table 1). Data losses were mostly the result of power failures unnoticed by the study participants. Data from three additional units from the first period were not recovered due to instrument failure, which required a reset of the units and full data loss. 

Although low-cost monitors have been designed for indoor purposes, their outdoor use under adverse weather conditions (cold winter with frequent snow) in the housings resulted in good durability of sensing elements, stable sensor sensitivity, limited hardware issues, and consequent data loss. These monitors are therefore also suitable for outdoor use when installed in a waterproof enclosure.

### 3.2. Mean PM_2.5_ Concentrations

The mean PM_2.5_ concentrations at the Monroe County measured by the TEOM at the DEC site for the two heating periods were 8.0 ± 5.6 and 6.0 ± 4.7 µg/m^3^, respectively. The causes of the lower concentrations during the second period (74% of the first period concentration, Figure S2 in the Supplementary Materials) are unknown. During the second period, there were higher ambient temperatures and thus a lower need for household heating. There was also higher precipitation during the second period, which would remove PM via wet deposition.

A similar decrease in concentrations (71% of the first-period concentrations observed during the second period) was observed in the Speck data, calculated as the average value over all units. The absolute values measured by Specks (mean PM concentrations of 3.5 ± 0.9 and 2.4 ± 0.4 µg/m^3^ during the first and second period, respectively) are lower than those measured by the TEOM. In both periods, the Specks measure about 40% of TEOM values (43% and 41%, respectively). The underestimation in bias-corrected Speck results suggests the bias corrections were too large, resulting from a different reference unit for the collocation than the TEOM. The comparison between the instrument used for the bias estimation, the Grimm 1.109, and the TEOM, showed a 55% overestimation by the Grimm compared to the TEOM, similar to the prior study [18]. Since the Speck underestimation compared to TEOM was the same for the two periods, the original bias corrections were applied, and relative metrics were used for the comparisons.

Taking the bias into account, the TEOM data were compared to Speck data using the Wilcoxon signed-rank test [23]. The null hypothesis was that the difference between each pair of datasets would be symmetrical about the difference of mean values of the corresponding datasets, i.e., the datasets are shifted by a constant value (mean TEOM—mean Speck value). The results differed by averaging time and measurement period. For the daily average values, more Speck–TEOM pairs showed statistically similar results compared to hourly averaged values (Table 2). This result suggests that daily averaging reduces some of the random variation in the Speck data. During the second measurement period, there were more similar Speck–TEOM pairs than during the first period. For both periods, however, the most similarities between TEOM and Speck data were found in daily data corrected on bias and LOD for Specks located in the city center (Table 2). If the constant shift between TEOM and Speck data was not considered, independently on averaging time, period, or location of Speck, none of the Specks was measuring data from an identical data distribution as TEOM.

### 3.3. Temporal Correlations

#### Speck vs. TEOM Correlations

**Figure 2:**
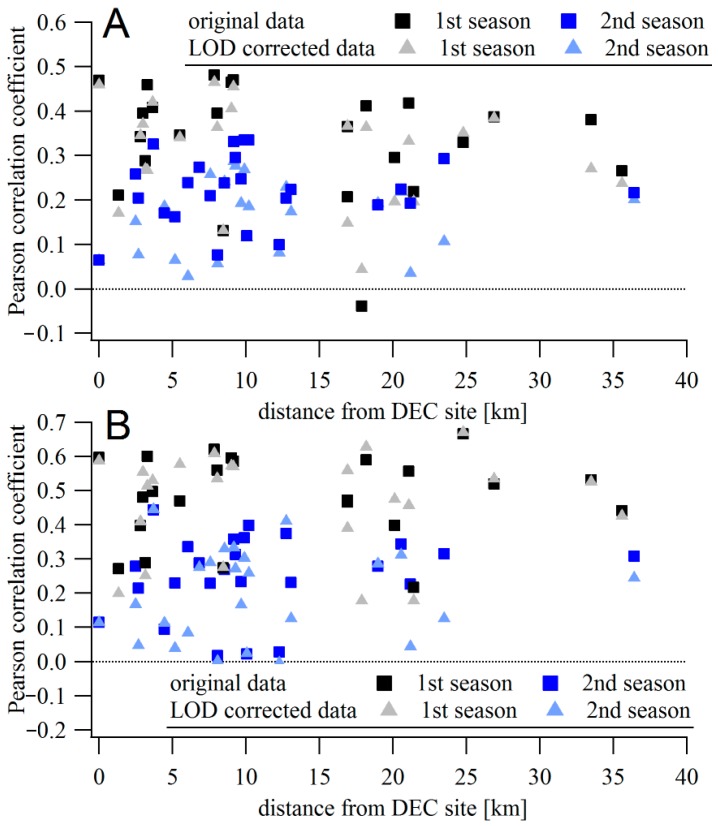
(**A**) Pearson correlation coefficient between hourly averaged concentrations measured by Speck units and TEOM located at the Rochester DEC site, dependent on the distance between Specks and the DEC site. (**B**) The same for daily averages.

Hourly and daily mean TEOM concentrations were compared to averaged data from the Speck monitors. The Speck data were calculated either from the original data or from data with values below LOD replaced with LOD/2 values. Figure 2 shows the correlation coefficients between the hourly- and daily-averaged concentrations measured by the Specks and the TEOM. The Speck units are arranged on the x-axis to show the distances from the DEC site. The mean correlation between hourly concentrations differed between the two periods. For the 2015–2016 measurement period, the mean Pearson correlation coefficient was 0.34 (Figure 2a). However, the mean correlation coefficient was only 0.21 during the second measurement period. The lower correlation may be due to the lower absolute values measured during the second period, resulting in measurements that are below the LOD and increasing the relative importance of local sources (e.g., vehicles, wood combustion) in the vicinity the individual monitors. The dependence of the correlation coefficient on the distance from the DEC site is not significant during either of the periods (slope of −0.002 ± 0.003 and −0.001 ± 0.003 during the first and second periods, respectively). For data with values below LOD replaced by the LOD/2 value, the mean correlation is comparable to the original value (0.31 for the first period and 0.16 for the second period). The dependence of correlation on the distance is also comparable, with slopes of −0.003 ± 0.002 and −0.001 ± 0.003 during the two periods. 

**Figure 3:**
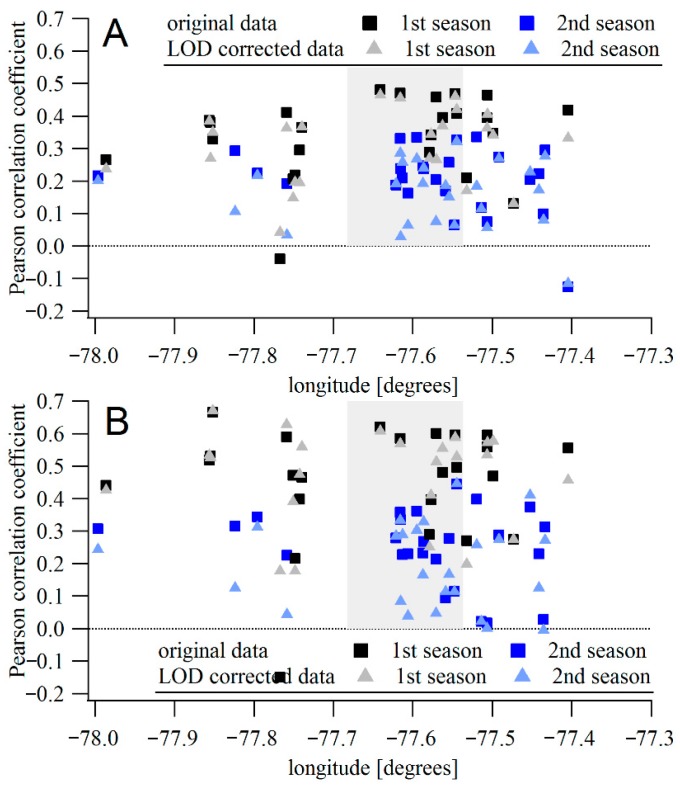
(**A**) Pearson correlation coefficient between hourly averaged concentrations measured by Speck units and TEOM located at the Rochester DEC site, dependent on the longitude of the Speck location (DEC site at −77.55°). The gray area highlights the Rochester city center. (**B**) The same for daily averages.

When the direction of the Speck monitor is considered, some dependence was found in the data. The mean correlation coefficient between DEC-site TEOM and Specks outside the city is lower than between the TEOM and those monitors located within the city (estimated as the area inside the RT590/RT390 highway loop), 0.31 vs. 0.39 during the first period and 0.19 vs. 0.24 during the second period. Those units outside the city on the west side (upwind of the city based on prevailing wind direction) show even lower correlations (0.28 in the first, and 0.17 during the second period) with the TEOM located in the city. The few Specks east of the city (downwind of the city based on the prevailing wind direction) show correlations comparable with those in the city. However, the distances between monitors east of the city and the TEOM are smaller than for the units west of the city (Figure 3a).

For daily averages, the mean correlation between TEOM and Specks is higher during the first period: 0.46 for all data (Figure 2b) and 0.47 with data below LOD replaced by LOD/2. Some of the units had correlation coefficients >0.6. During the second period, the daily values show lower mean correlation coefficients; 0.23 for all data considered and 0.18 for data with the data below LOD replaced. The correlation coefficient did not depend on the distance from the DEC site (slope of 0.001 ± 0.003 and −0.001 ± 0.004 for the original data, 0.002 ± 0.004 and −0.001 ± 0.004 for the LOD-corrected datasets in respective periods).

The highest correlation coefficients with the TEOM were for Specks within the city (mean value 0.48) while Specks outside the city show mean correlation coefficient of 0.44 (Figure 3b). During the second period, the mean correlation between Specks in the city (0.25) is also higher than outside the central region (0.21). However, if only the units located to the west are considered, their mean correlation coefficient with TEOM is higher, 0.30. There were only four units located to the west, compared to 11 in the central city area. The correlation coefficients across the county are lower than those calculated from the side-by-side collocations with the Grimm instrument (mean correlation coefficients of 0.61 and 0.77 for hourly data during the first and second collocation experiments, respectively), suggesting the lower correlations come from real differences in the concentrations, and not from limitations of the monitors. Multiple distributed PM sources as well as differences in deposition, dilution, and physicochemical transformations of the aerosol across the city result in high spatial–temporal variability [5,19]. The DEC site is close to major roads, the railroad, and residential areas with substantial recreational wood combustion [20,21] that would not necessarily covary with other areas of the measurement domain. Therefore, a wide range of correlations with the TEOM is not surprising.

### 3.4. Correlation Matrixes

**Figure 4:**
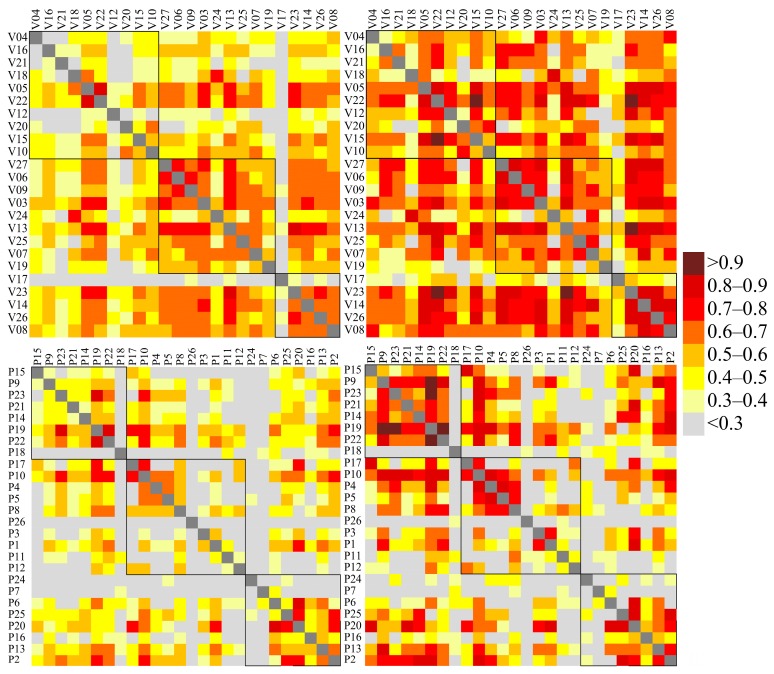
Pearson correlation coefficient between Speck PM monitors calculated from hourly (**left**) and daily (**right**) PM averages, and from the 1st period (**top**) and 2nd period (**bottom**). LOD uncorrected data only were considered. The black squares denote groups of measurement sites geographically similar to each other.

For the Speck monitor data only, Pearson correlation matrices were calculated based on hourly and daily data, from both the original and LOD-corrected dataset. The data in correlation matrices are presented according to Speck location, dividing the Speck monitors into three groups: Specks located west of the city center; in the city center; and east of the city center. The division was based on the prevailing wind direction, which was westerly during both years (wind roses are presented in Figure S3). In Figure 4, the three groups are highlighted with three squares. The numbering was given to Specks randomly during the installation and a close number does not mean a close position between the two units. The individual Speck positions are presented in Figure 1.

The weak correlations observed during the second period between the Speck and TEOM data can be observed in the correlation matrixes (Figure 4). During the first period, 28% of the Speck pairs showed correlation >0.6 and almost 50% of the pairs show correlation >0.5 (Figure 4 and Table 3). Within the three groups, the largest variability in hourly correlation coefficients was found in the western group, where only the units located close to each other showed high correlations (for example V05 and V22). For most units, the correlation was <0.5, resulting in a mean correlation coefficient within the group of 0.41, probably due to the large distances between units, and limited PM sources, most of which would be local. In the city center, the distances between Speck units were smaller and with higher local source emissions, such as traffic, resulting in higher correlation coefficients. Approximately 20% of the coefficients in the city center are >0.7, and 55% are >0.6, with mean value of 0.6. Except for V17, which showed correlation coefficients <0.4, correlations >0.6 were observed between all units in the eastern group. Correlations outside the three groups were generally lower. In the dataset with data below LOD replaced, the correlations of hourly averages decreased (only 10% show correlation >0.6). The largest decrease was observed between the units with a low fraction of data above the LOD.

During the second period, only 10% of the pairs showed correlations >0.6 (Table 3). For these units, the mean correlations were similar (~0.4) in all three groups. In all three groups, several units showed correlations <0.3 with any other unit (Figure 4). The difference from the first period may be explained by different locations of the units, different distances between the units, and/or by the lower mean concentrations during the second period. 

Comparing hourly and daily data, higher correlation coefficients were found for the daily data for both periods. During the first period, more than half of the correlation coefficients were >0.6, while during the second period almost 30% of pairs showed correlations >0.6. The higher correlation coefficients were found both within the three groups but also between sites across the groups. The patterns in the correlation matrix are similar for the daily data, with mean correlations of 0.5 in the western group, 0.6 in the city center, and 0.7 in the eastern group for the first period. The effect of well-mixed pollution from the city influencing downwind locations may have produced the high correlations in the eastern group. In the second period, the highest mean correlation was found in the western group (0.5), while in the city center and eastern part correlations of 0.4 were found.

### 3.5. Coefficients of Divergence

The coefficients of divergence (COD) were calculated for the bias corrected hourly and daily datasets, including both the LOD corrected and uncorrected datasets. The COD values between TEOM concentrations and individual Speck PM concentrations were compared to the position of instruments.

**Figure 5:**
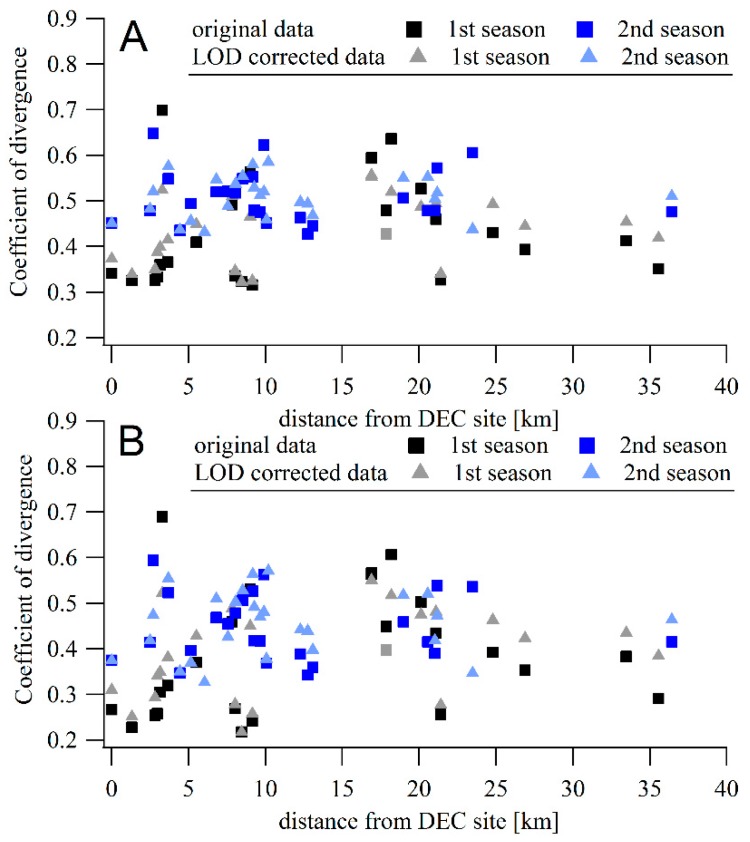
(**A**) COD between hourly averaged concentrations measured by Speck units and TEOM located at the Rochester DEC site, dependent on the distance from the DEC site. (**B**) The same for daily averages.

A decrease in COD for Speck monitors located close to the DEC site where the TEOM was located is visible in Figure 5 for the first period. Comparing hourly and daily averages, lower COD values were found for daily data, with COD between TEOM and the closest Specks almost 0.2 (showing a homogeneity in data). The mean COD value between TEOM and Speck in the city center is 0.36 (including V13 showing COD of 0.69, excluding this unit, the mean COD decreases to 0.32) for daily data, and 0.41 for hourly data (0.36 without V13 unit). If the LOD-corrected datasets are considered, an increase of 0.04 was observed in both hourly and daily data. The mean COD of Specks in regions outside the city center are higher than those in the city center, 0.44 and 0.37 for daily data from the west and east, respectively, and 0.48 and 0.42 for comparable hourly averages. Outside the city center, no change in mean COD values was observed between LOD corrected and non-corrected datasets.

The COD was also calculated between the Speck units and presented in a matrix, similarly to the analyses of correlation coefficients. The COD values were calculated for hourly and daily averages from bias-corrected PM concentrations, both from LOD-corrected and uncorrected datasets. The Speck units were again grouped according to their geographical positions into three groups—west, center, and east.

For the hourly data, 20% of the pairs showed COD < 0.2 (Table 4), suggesting homogeneity in the data. Most of the pairs, however, show COD between 0.2 and 0.4. For the daily data, homogeneity was found in 37% of pairs. For the LOD-corrected data, the ratio of COD below 0.2 increases for hourly and daily datasets as well, and no pair shows COD > 0.6.

**Figure 6:**
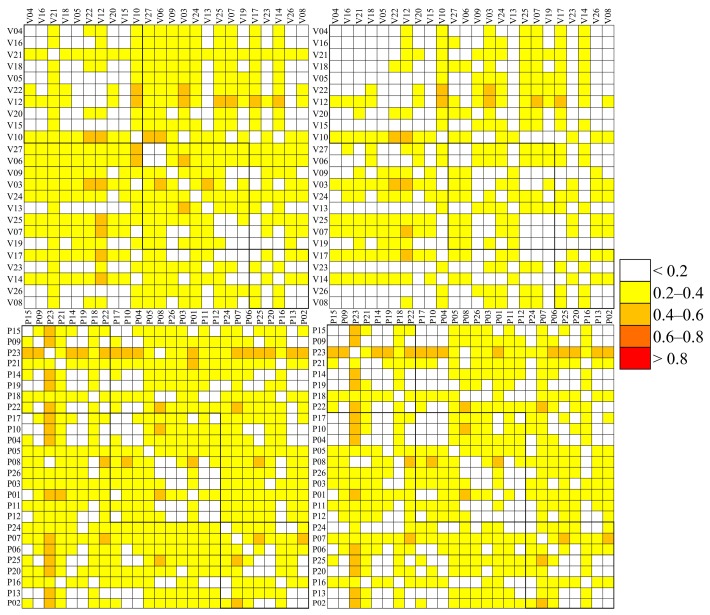
Top: COD between Speck PM monitors calculated from hourly (**left**) and daily (**right**) PM averages from bias and LOD corrected data from the first campaign, bottom: the same for the second campaign.

If the units from the three groups (denoted in Figure 6 as thick squares) are considered separately, the mean COD decreased to at or below 0.2 for the LOD-corrected data outside the city center (Table 5), suggesting homogenous concentrations within the groups. The mean COD values in individual groups are not the same, but there is no statistically significant difference between the groups (evaluated by Dunn’s test [29]).

### 3.6. Spatial Interpolation

Spatial interpolation assumes that spatially distributed objects are correlated. The PM concentrations at unmeasured locations are predicted from the limited number of sampled location (the participant houses, in this case) using functions to estimate the correlations within the measurement domain (Monroe County). For such analyses, increasing the density of data (sampling sites) will generally produce more accurate estimations. Hourly and daily average concentrations collected during the 1st and 2nd heating periods were merged and jointly used as input for IDW spatial interpolations (power 2). Since the PM concentrations measured at the reference site (DEC) were statistically different between the two periods (Kruskal–Wallis analysis of variance at *p* < 0.05), the average concentrations calculated over the second period were scaled to harmonize the dataset. In this case, the 2nd-year concentrations were multiplied by 1.35 (i.e., the ratio between mean PM_2.5_ concentration during the first period to mean PM_2.5_ concentration during the second period as measured by TEOM) to obtain comparable concentrations.

**Figure 7:**
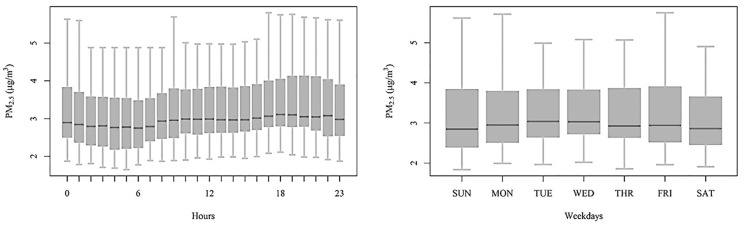
The mean daily and weekday patterns of the PM concentrations measured over the two periods.

**Figure 8:**
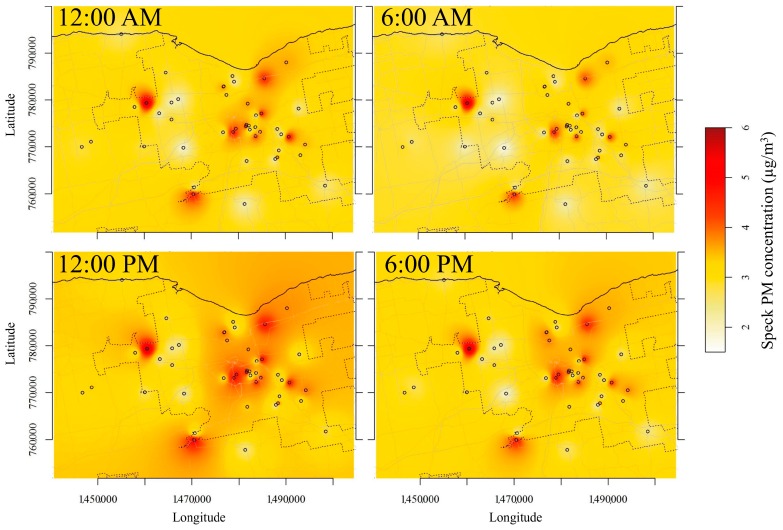
Hourly spatial distributions at the county at midnight, 6 a.m., noon, and 6 p.m., averaged over the two periods.

The daily and weekday patterns of the PM concentrations measured over the two periods are reported in Figure 7. The resulting maps of the hourly and weekday spatial interpolations are shown as animations in Figures S4 and S5 in the Supplementary Materials, respectively, while Figure 8 summarizes the hourly spatial distributions at midnight, 6 a.m., noon, and 6 p.m. Generally, the daily pattern of PM shows minimal concentrations over all the sites at 5–6 a.m., followed by an increase until noon at several sites due to the morning rush hour. The PM concentrations maintain stable concentrations until 3 p.m., when a second peak occurs and raises the PM concentration to maximum levels around 6 p.m., i.e., during the evening rush hour. The weekly pattern shows minimum concentrations during the weekends and the maximum on Tuesdays and Wednesdays. The daily and weekly patterns of most Speck units agree well with the patterns of PM_2.5_ measured by FEM at the DEC site between 2004 and 2015 [22].

The daily and weekly patterns are influenced by both human activities and meteorological conditions. Although explicit source apportionment methods were not applied, some insights into the most relevant emission sources can be hypothesized based on the observed diurnal patterns. Road traffic volume for the major roads in Rochester during winter were provided by the NYS Department of Transportation, which performed hourly traffic counts during two weeks in December 2015 and January 2016. Figure S6 shows the hourly and weekly traffic count patterns that exhibit typical morning and evening rush hour peaks. This profile relates well with the Speck PM profile (Figure 8). Thus, road traffic is one of the more influential PM sources. The concentration decreases during the nighttime, which suggests the limited effect of domestic heating emissions on PM concentrations. Most participants reported that they mostly use their wood stoves on the weekends for recreational purposes. Since the lower concentrations are recorded during the weekends, local wood emissions does not seem to have had a major impact on PM concentrations. The diel pattern also shows that the mixing layer dynamics have a limited effect on PM concentrations. The lowest PM concentrations were recorded at 6 a.m., i.e., during the coldest part of the day when the mixed layer reaches its lowest height. The lower concentrations during the late afternoon rush hour compared to the morning values suggest some effect of the rising mixed layer heights.

The interpolated maps also show that higher PM concentrations on both hourly and weekday bases were generally recorded in the city of Rochester. However, there is a large spatial variation of PM concentrations, which is likely related to local emission sources. In addition, high concentrations were also found near the village of Parma (west of Rochester) and Scottsville (southwest of Rochester). These results confirm the high spatial variability of PM concentrations across the county. This spatial resolution obtained from a single monitor is therefore insufficient to capture the spatial variability required to accurately represent human exposure for use in epidemiological studies, similar to what had been reported by Wang et al. for wood smoke [19] and ultrafine particles [32]. Thus, the use of low-cost monitors can be a useful option to reduce exposure misclassification. The Speck data will be used for modelling the spatial variations of PM through the implementation of an hourly land use regression model in a future paper.

### 3.7. CBPF

The CBPF was calculated from hourly data for PM_2.5_ concentrations measured at the DEC site by TEOM and at the locations measured by Specks as well, separately for each of the periods. The wind speed and direction data from the Greater Rochester International Airport were used as representative of the meteorological conditions across the measurement domain. The CBPFs from Speck monitors were calculated from averaged data. The mean hourly concentrations from each geographical group of sensors (western, center, and eastern) were compared to wind data to estimate the influence of wind speed and direction on measured concentrations. Furthermore, the result was compared to CBPF from TEOM data measured at the DEC site (located in the city center).

**Figure 9:**
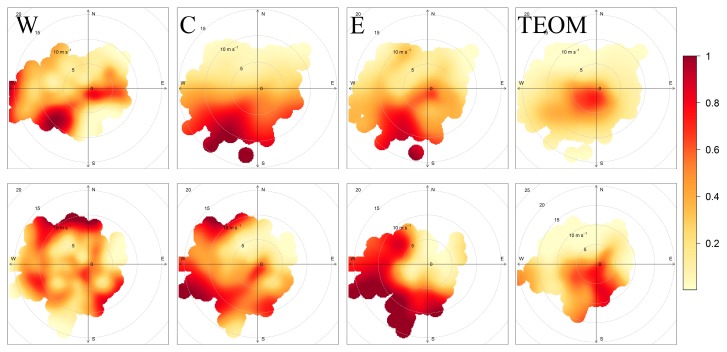
Top: Conditional bivariate probability function (CBPF) calculated from mean Speck values in the three groups of units: western (W), city center (C), and eastern (E), compared to CBPF from TEOM data. Calculated from the first period data. Bottom: The same for the second period.

The CBPF calculated from TEOM data shows that the highest probabilities of the concentrations being higher that the median values (6.8 µg/m^3^ and 4.9 µg/m^3^ during the first and second period) were under low wind speeds with the main contribution from local sources (Figure 9). A similar result was found for the Specks located in the city center. However, the Specks showed some sources connected to higher wind speeds and SW wind directions. This result may reflect the multiple locations of the units compared to the DEC site.

In the western group, stagnant conditions also proved to be the periods with higher concentrations. Additional sources were located east of the units, suggesting the city as a source of PM in this area. Sources were found during periods with wind speeds >15 m/s, with resuspension or distant sources playing their role. In the eastern group, stagnant conditions were found to be related to high measured PM concentrations during the first period, with an additional source located SW of the region, pointing again to the Rochester city center. During the second year, however, the main contribution was found for sources located outside the region (W and SW of the area).

## 4. Conclusions

Clearly, these monitors would perform better in environments with higher concentrations. At the ambient concentrations in Rochester, there are issues of accuracy for these low-cost monitors given the need for large bias corrections. However, their precision as reported by Zikova et al. [18] as 12% for outdoor data, is sufficient to permit the determination of the spatial and temporal patterns of PM across an extended area over multiple months. They worked over two separate seasons with limited problems. Thus, these monitors provide the opportunity for much more intensive monitoring networks based on a central site monitor where high-accuracy PM data are being collected. They could be used to identify high concentration areas or provide very detailed monitoring within a smaller area. The relationships between measured values and locations, time of day, day of week, and meteorological conditions were reasonable. Thus, these data can serve as the basis for further modeling to provide more accurate exposure assessments for future work on the relationships between PM exposure and various possible adverse health outcomes.

## Figures and Tables

**Table 1:** Number of measured data points (each corresponding to a 1-min measurement), data coverage of the 50 low-cost measurement units, percentage of data over LOD, and a bias correction factor. Units’ names starting with V denote units measuring during the first year, names starting with P units measuring during the second year.

Unit #	# Data Points	Coverage [%]	>LOD [%]	Corr. Factor	Unit #	# Data Points	Coverage [%]	>LOD [%]	Corr. Factor
V03	171319	100	83.2	0.41	P01	227379	100	20.0	0.18
V04	172430	100	16.3	0.47	P02	224369	100	51.0	0.20
V05	169578	99.5	5.5	0.40	P03	225808	100	39.7	0.22
V06	172710	100	34.7	0.29	P04	215369	95.9	13.0	0.29
V07	84731	92.3	81.6	0.41	P05	224224	100	58.1	0.25
V08	173872	100	4.3	0.53	P06	224292	100	37.8	0.27
V09	172299	100	51.4	0.41	P07	214569	100	66.7	0.27
V10	185767	100	82.8	0.41	P08	150726	66.7	0.8	0.72
V12	171244	100	15.2	0.31	P09	222425	100	18.6	0.28
V13	171412	100	1.8	0.41	P10	138294	63.8	15.9	0.25
V14	172625	100	90.3	0.34	P11	220149	98.6	95.3	0.24
V15	173688	100	4	0.46	P12	224304	100	55.8	0.36
V16	170445	100	3.8	0.52	P13	220931	100	19.6	0.29
V17	99435	57.8	100	0.30	P14	221274	99.2	55.0	0.21
V18	172537	100	17.3	0.47	P15	202146	98.3	63.3	0.25
V19	175178	92.9	3.7	0.67	P16	226502	97.1	64.5	0.27
V20	106170	94.2	0.4	0.59	P17	212246	97.3	59.4	0.26
V21	172716	100	39.9	0.3	P18	217567	100	90.9	0.24
V22	128105	71.3	13	0.35	P19	111800	100	12.7	0.34
V23	174482	100	4.8	0.49	P20	102458	98.9	79.9	0.16
V24	170041	100	56.2	0.39	P21	216764	99.1	1.5	0.56
V25	180491	100	74.9	0.36	P22	216647	99.9	3.7	0.27
V26	185397	100	8.9	0.44	P23	214108	99.2	1.7	0.96
V27	181548	100	44.7	0.27	P24	187046	100	27.0	0.33
					P25	159439	99.8	67.5	0.15
					P26	200203	91.5	99.9	0.17

**Table 2:** Number of TEOM–Speck monitors data pairs (out of 24 and 26 during the first and second period) coming from identical populations, according to paired Wilcoxon Signed-Rank Test. Numbers in brackets show the number of (TEOM–Speck) pairs located in the city center.

	Hourly	Hourly LOD	Daily	Daily LOD
First period	2 (1)	2 (1)	2 (1)	8 (5)
Second period	4 (2)	4 (2)	11 (4)	11 (5)

**Table 3:** Percentage of Pearson correlation coefficients in the correlation matrixes of Speck PM concentrations calculated from hourly and daily averages, from the original and LOD corrected data (denoted as LOD in the table).

	Correlation Coefficient	>0.9	>0.8	>0.7	>0.6	>0.5	>0.4	>0.3
First period	hourly	0.0	0.7	7.2	28.3	48.9	68.5	85.1
	hourly LOD	0.0	0.4	2.5	10.9	27.5	43.5	64.1
	daily	1.1	13.0	31.2	54.7	73.2	84.4	93.8
	daily LOD	0.7	8.0	23.9	41.7	56.9	72.5	85.5
Second period	hourly	0.0	0.9	4.6	10.2	26.2	44.3	57.5
	hourly LOD	0.0	0.9	2.2	8.3	14.8	26.5	36.0
	daily	0.9	7.1	16.0	27.7	37.5	52.3	62.2
	daily LOD	0.0	0.0	1.8	6.8	12.9	22.8	35.1

**Table 4:** Percentage of COD values between each Speck PM concentration pair calculated from hourly and daily averages, from the original and LOD corrected data (denoted as LOD in the table).

	COD Values	Hour	Hour LOD	Day	Day LOD
First period	<0.2	22.8	30.8	37.3	50.0
	0.2–0.4	49.3	64.9	46.0	47.8
	0.4–0.6	24.6	4.3	15.6	2.2
	0.6–0.8	3.3	0	1.1	0
	>0.8	0	0	0	0
Second period	<0.2	25.5	23.1	33.2	35.4
	0.2–0.4	37.8	69.8	43.4	58.5
	0.4–0.6	22.5	7.1	13.8	6.2
	0.6–0.8	13.8	0	9.5	0
	>0.8	0.3	0	0	0

**Table 5:** Mean COD values between each Speck PM concentration pair calculated from hourly and daily averages, from the original and LOD corrected data (denoted as LOD in the table) in the three separate geographical groups. In the city center, the mean value from all pairs, with the exclusion of V13, is presented (in the brackets).

	Group	Hour	Hour LOD	Day	Day LOD
First period	West	0.24	0.17	0.31	0.20
	Center	0.24 (0.16)	0.21 (0.19)	0.28 (0.20)	0.24 (0.22)
	East	0.21	0.18	0.25	0.19
Second period	West	0.34	0.22	0.26	0.19
	Center	0.32	0.28	0.28	0.24
	East	0.28	0.21	0.23	0.19

## Supplementary Materials

The following are available online at http://www.mdpi.com/1424-8220/17/8/1922/s1, Figure S1: Inside of outdoor housing for Speck monitor with labeled inlets, Speck monitor, and a bulb for heating, figure S2: Top: Hourly TEOM PM_2.5_ concentrations from the Rochester DEC site, and mean PM concentrations as measured by Speck monitors during the first period. Bottom: The same for the second period. Figure S3. Wind roses from hourly data from the first (left) and second (right) period. Figure S4. Hourly IDW spatial interpolations of the PM concentrations across the county calculated from two measurement periods. Figure S5. Daily IDW spatial interpolations of the PM concentrations across the county calculated from two measurement periods. Figure S6. Daily and weekly patters of road traffic measured on a major road in Rochester between December 2015 and January 2016. Data provided by NYS DOT. There are also two movies showing hourly and day of week variations in PM concentrations across the measured domain.
