# Supplementary material for: Estimating Hourly Concentrations of PM2.5 across a Metropolitan Area Using Low-Cost Particle Monitors
Source: Sensors (Basel). 2017 Aug 21;17(8):1922. doi: 10.3390/s17081922 (PMC5579734; doi:10.3390/s17081922)
Supplement: Supplementary file 1 [file sensors-17-01922-s001.pdf]

Estimating Hourly Concentrations of $PM_{2.5}$ across a Metropolitan Area Using Low-Cost Particle Monitors

Nadězda Zíková [1,2], Mauro Masiol [2,3], David C. Chalupa [4], D. Q. Rich [3], Andrea R. Ferro [5], Philip K. Hopke [2,3*]

1 Institute for Environmental Studies, Faculty of Science, Charles University, Prague 12801, Czech Republic; nada.zikova@natur.cuni.cz
2 Center for Air Resources Engineering and Science, Clarkson University, Potsdam, NY 13699, USA; mmasiol@clarkson.edu
3 Department of Public Health Sciences, University of Rochester School of Medicine and Dentistry, Rochester, NY 14642, USA, David_Rich@urmc.rochester.edu
4 Department of Environmental Medicine, University of Rochester Medical Center, Rochester, NY 14642, USA; David_Chalupa@urmc.rochester.edu
5 Department of Civil and Environmental Engineering, Clarkson University, Potsdam, NY 13699, USA; aferro@clarkson.edu
* Correspondence: phopke@clarkson.edu; Tel.: +1-585-276-3240

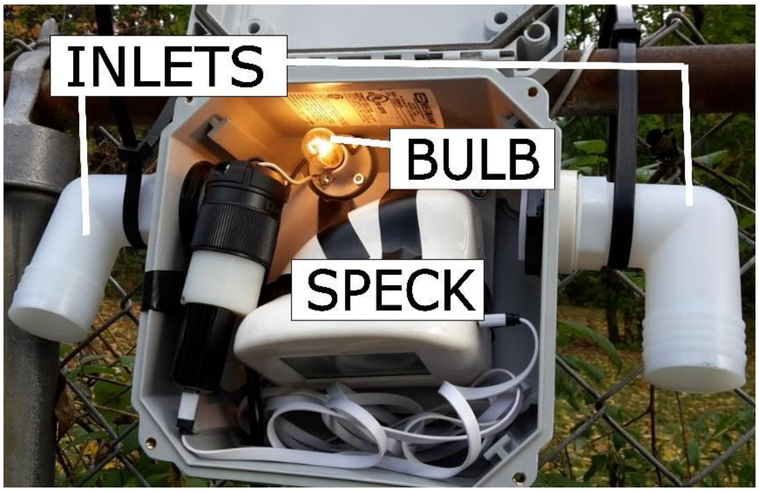

Figure S1. Inside of outdoor housing for Speck monitor with labeled inlets, Speck monitor, and a bulb for heating.

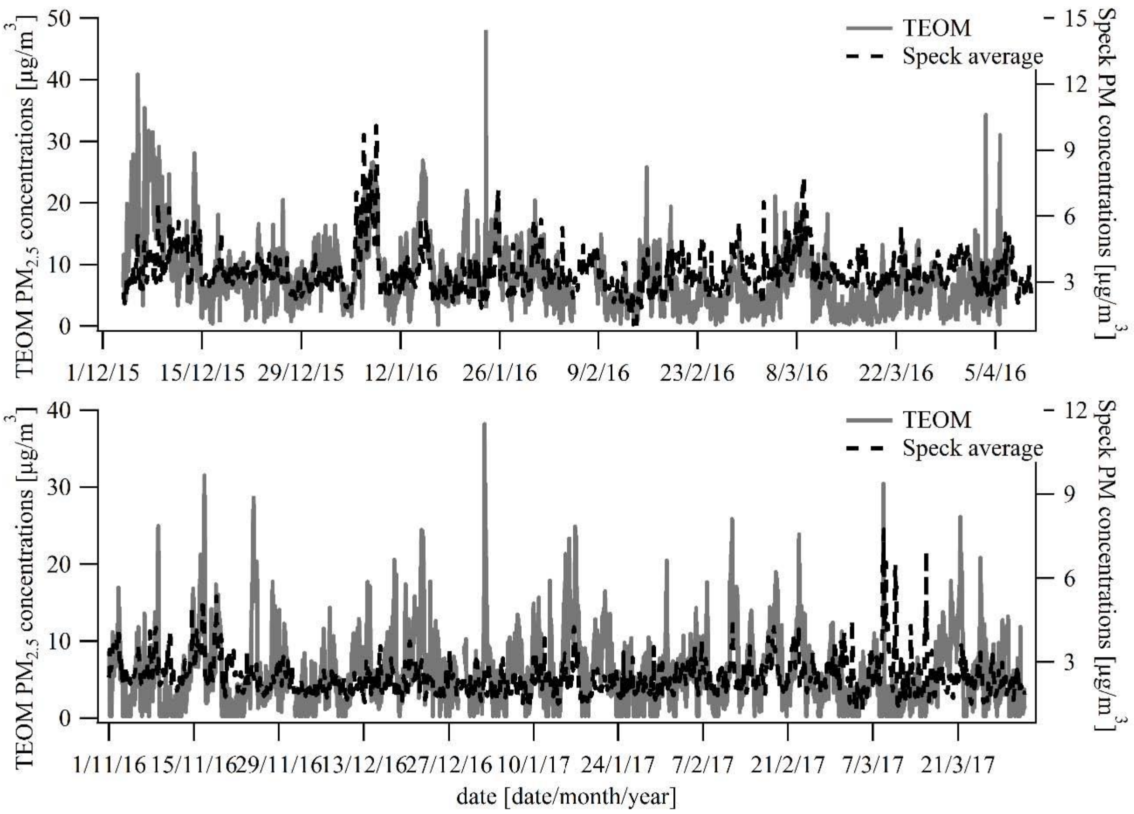

Figure S2. Top: Hourly TEOM $PM_{2.5}$ concentrations from the Rochester DEC site, and mean PM concentrations as measured by Speck monitors during the first season. Bottom: The same for the second season.

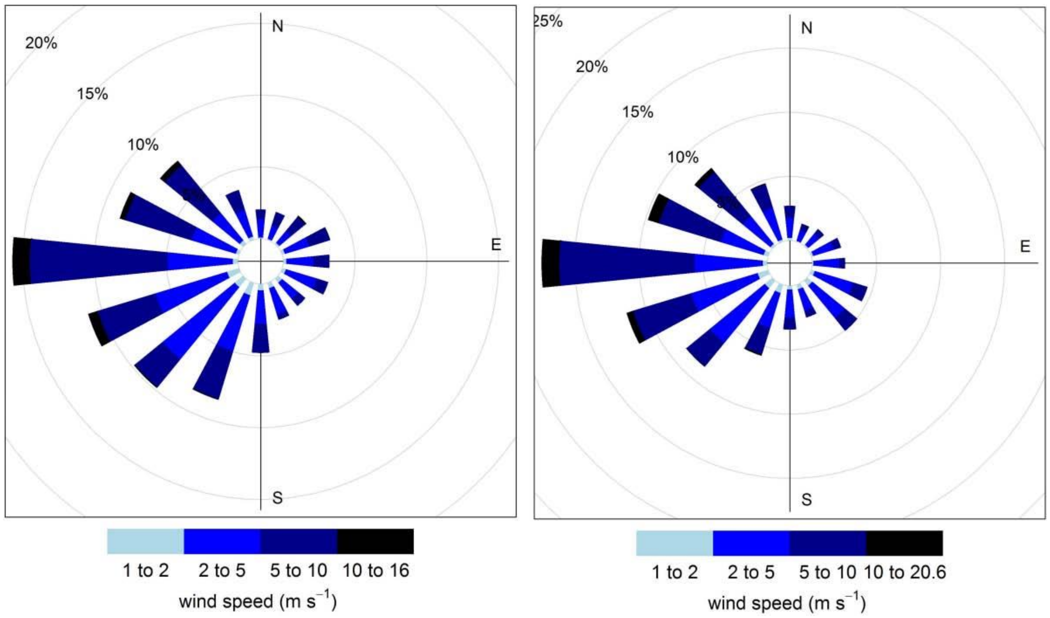

Figure S3. Wind roses from hourly data from the first (left) and second (right) season.

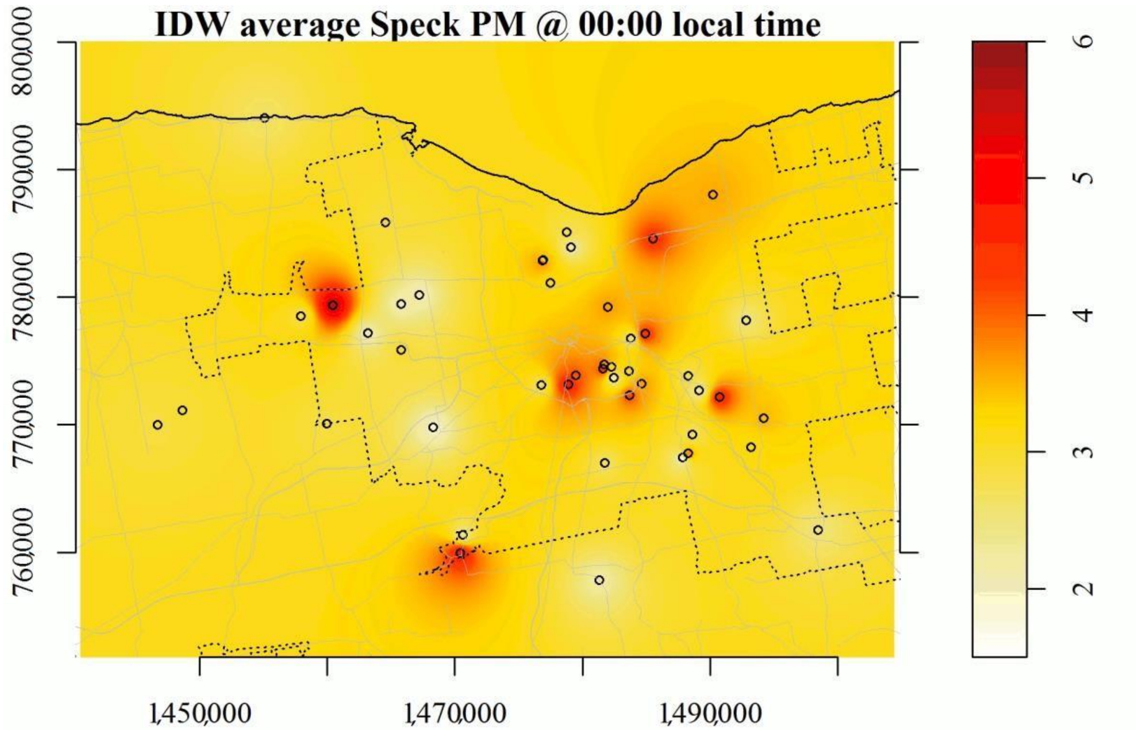

Figure S4. Hourly IDW spatial interpolations of the PM concentrations across the county calculated from two measurement seasons.

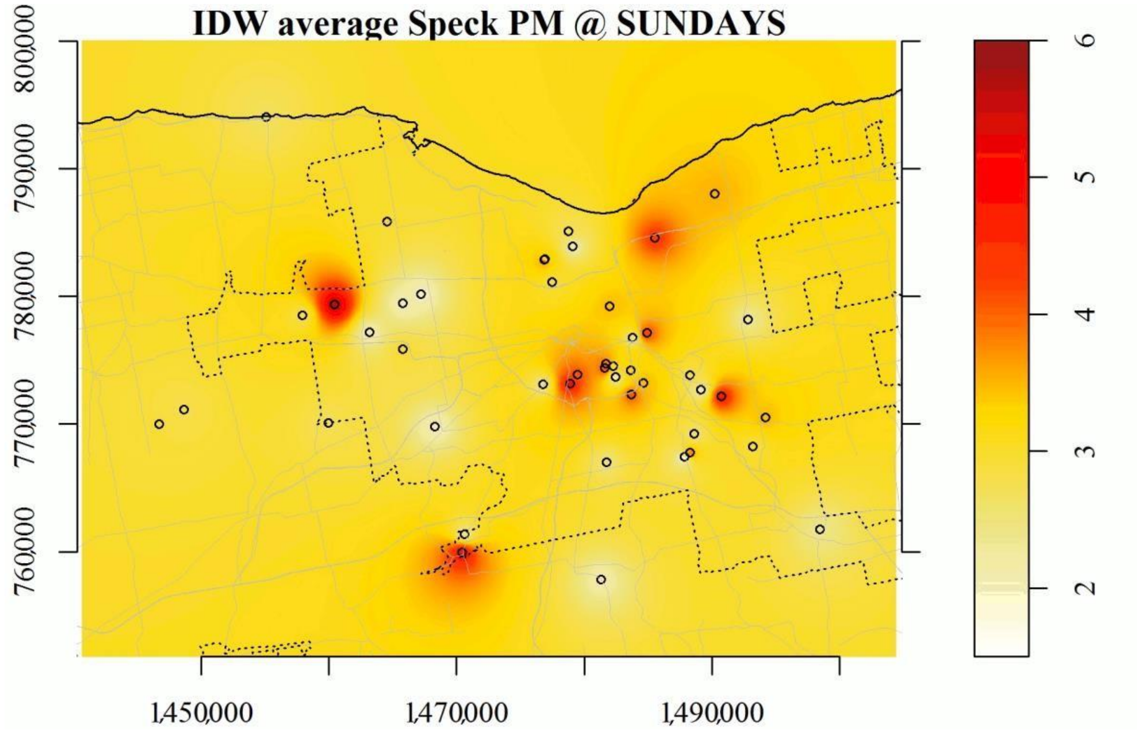

Figure S5. Daily IDW spatial interpolations of the PM concentrations across the county calculated from two measurement seasons.

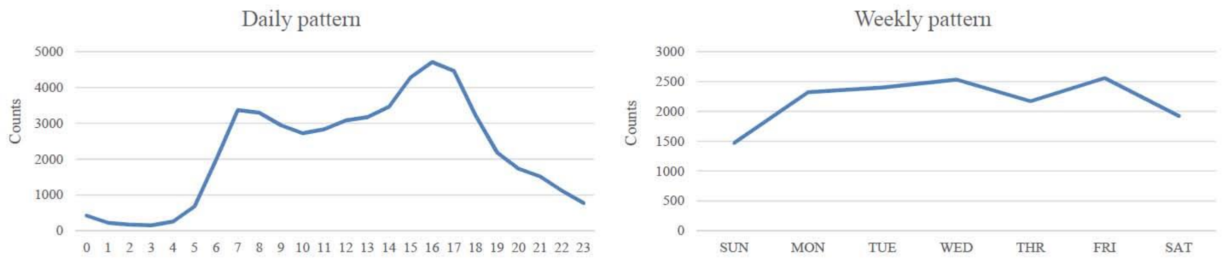

Figure S6. Daily and weekly patters of road traffic measured on a major road in Rochester between December 2015 and January 2016. Data provided by DoT.
